# Prognostic Significance of Left Atrial Size in the Recurrence of Atrial Fibrillation

**DOI:** 10.7759/cureus.100449

**Published:** 2025-12-30

**Authors:** Marina Katerini, Christine Politi, Eleni Kyritsi, Eugenia Minasidou, Lambrini Kourkouta, Konstantinos Koukourikos, Areti Tsaloglidou

**Affiliations:** 1 Cardiology, General Hospital of Edessa, Edessa, GRC; 2 Nursing, University of West Attica, Athens, GRC; 3 Nursing, International Hellenic University, Thessaloniki, GRC

**Keywords:** atrial fibrillation, echocardiography, left atrial diameter, recurrence, risk stratification

## Abstract

Introduction: Atrial fibrillation (AF) is associated with significant morbidity and recurrence despite rhythm control strategies. Identifying reliable predictors of AF recurrence is essential for optimizing patient management.

Aim: The aim of this study was to evaluate the predictive value of left atrial (LA) diameter for AF recurrence at six and 12 months following rhythm control interventions.

Methods: This prospective observational study included 66 patients with documented AF who underwent rhythm control interventions, including electrical or pharmacological cardioversion. LA diameter was measured by transthoracic echocardiography in the parasternal long-axis view at end-systole, using a standardized protocol by a single blinded operator. LA volume and indexed measurements were not consistently available and were excluded. The primary endpoint was AF recurrence at six and 12 months. AF recurrence was defined as any documented AF episode lasting >30 seconds, confirmed by 12-lead ECG during scheduled visits or symptom-driven ECG recordings. Follow-up was conducted at six and 12 months. Two patients were lost to follow-up.

Results: The study included 66 patients (71.2% men) with a mean age of 65.4 ± 13.7 years. Hypertension (74.2%) and dyslipidemia (54.5%) were the most prevalent comorbidities, while 28.8% were smokers. Mean left ventricular ejection fraction was 52.3 ± 10.5%, with 77.3% of patients having preserved systolic function (LVEF ≥ 50%). Mean left atrial diameter was 43.5 ± 7.2 mm; 31.8% had LA diameter <40 mm, and 12.1% >50 mm. Rhythm control was achieved using electrical cardioversion or antiarrhythmic drugs, with palpitations being the most common presenting symptom. AF recurrence occurred in 51.6% of patients at six months and 50.8% at 12 months. Background pharmacotherapy remained stable during follow-up. LA size >42.5 was significantly associated with having an AF episode at six (OR=7.55; 95%CI: 2.22 - 25.66; p=0.001) and 12 months (OR=41.97; 95%CI: 7.37 - 239.07; p<0.001), after adjusting simultaneously for age, gender, smoking, alcohol, and number of comorbidities. Also, patients who smoked had a significantly greater probability of having an AF episode at 12 months (OR=12.9; 95%CI: 1.70 - 97.75; p=0.001). All possible interactions were tested, but no significant results emerged. The change in AF type distribution (from paroxysmal to persistent) between consecutive timepoints was not significant (p>0.05).

Conclusions: LA diameter >42.5 mm is a simple, non-invasive, and powerful predictor of AF recurrence after rhythm control. Its routine assessment may enhance early risk stratification and guide individualized therapeutic strategies in patients with AF.

## Introduction

Atrial fibrillation (AF) is the most common sustained cardiac arrhythmia globally and is associated with significant cardiovascular morbidity and mortality, largely due to its increasing incidence and high lifetime risk. Epidemiological data suggest that AF affects approximately 2-3% of the European population, with prevalence exceeding 5% among adults aged over 65 years [1,2].

The left atrium (LA) plays a fundamental role in maintaining normal cardiac physiology, functioning through three phases during the cardiac cycle. During ventricular systole, it acts as a reservoir by collecting blood from the pulmonary veins. In early diastole, it serves as a conduit, allowing passive blood flow to the left ventricle (LV). Finally, in late diastole, it functions as a booster pump, contracting to augment LV filling [3,4]. Both LA size and function have emerged as essential imaging biomarkers and provide prognostic information in various cardiovascular diseases such as AF, heart failure, mitral valve disease, and ischemic stroke [3-5]. LA enlargement is commonly observed in patients with AF and reflects chronic pressure or volume overload. It is closely related to the severity of LV diastolic dysfunction and serves both as a consequence and a promoter of atrial remodeling [6,7]. LA dilation enhances the structural and electrophysiological substrate for AF maintenance, creating a self-perpetuating cycle of atrial dysfunction, fibrosis, and electrical heterogeneity [8]. AF episodes themselves lead to impaired atrial contraction and elevated LA pressure, promoting further enlargement. This pathophysiological process contributes to increased thromboembolic risk due to blood stasis, particularly within the left atrial appendage [9]. Maintenance of sinus rhythm is therefore vital in reducing the risks associated with AF.

Assessment of LA size and function is typically performed via transthoracic echocardiography (TTE), due to its non-invasive nature, ease of use, and reproducibility. In selected cases, additional imaging modalities are also utilized. Cardiac magnetic resonance imaging (CMR) provides high-resolution, three-dimensional images and is considered the gold standard for assessing atrial volume and fibrosis [10]. Multidetector computed tomography (MDCT) provides detailed anatomical information, which is particularly valuable in procedural planning before catheter ablation. Transesophageal echocardiography (TEE) allows superior visualization of the left atrial appendage and interatrial septum, especially when evaluating for thrombus or guiding interventions [11].

Previous studies have reported inconsistent findings regarding the predictive value of LA size for AF recurrence, with some demonstrating a strong association [12], while others found limited prognostic utility [4]. These discrepancies are partly due to methodological limitations, such as small or heterogeneous study populations, retrospective designs, non-standardised echocardiographic measurements, and different definitions of AF recurrence. Although LA volume index and LA strain are considered more comprehensive markers of atrial remodeling, LA diameter remains the most widely used and accessible echocardiographic parameter in routine clinical practice. However, its prognostic role in predicting AF recurrence remains uncertain, particularly in real-world settings.

This study refers to secondary prevention and was designed to evaluate the association, rather than causality, between LA diameter, measured using a standardized TTE protocol, and the risk of AF recurrence at six and 12 months following restoration of sinus rhythm. All rhythm control interventions, including pharmacological and electrical cardioversion, were performed according to standard clinical criteria and current European Society of Cardiology (ESC) guidelines. As advanced LA functional parameters are not consistently available in many regional hospitals, it is important to assess whether LA diameter alone can reliably predict AF recurrence. 

Identifying whether LA diameter independently predicts AF recurrence could have significant clinical implications, including improved risk stratification, better-tailored rhythm control strategies and more targeted follow-up for patients at higher risk.

## Materials and methods

This prospective observational study was conducted at the Cardiology Department of the General Hospital of Edessa, Edessa, Greece, over a 16-month period, from December 2023 to March 2025. The study protocol was approved by the Scientific Council of General Hospital of Pella - Edessa Unit (application number: ES/10133/28-11-2023). The study was conducted in accordance with international ethical standards.

Study population and sample size

Inclusion criteria were admission with paroxysmal or persistent AF, availability of high-quality TTE images, and provision of informed consent for 12-month clinical follow-up. Exclusion criteria were permanent AF, severe structural heart disease, incomplete echocardiographic or clinical data, or poor acoustic windows that prevented accurate image acquisition.

It was calculated that with a sample size of 60 patients or more and assuming that almost half of the participants would experience an AF episode, the study would have 85% power to detect significant differences at an effect size of 0.8 or more. A total of 66 consecutive patients admitted with documented paroxysmal or persistent AF were enrolled. 

Data collection

Specifically, LA diameter was obtained in the parasternal long-axis view at end-systole, in accordance with ASE/EACVI recommendations. Measurements were performed by a single blinded operator to minimize inter-observer variability. LA volume index and LA strain were not included because they were not consistently available across all patients. Patients who underwent pharmacologic or electrical cardioversion were analyzed as a single group, as common selection and follow-up criteria were used. Patients who achieved spontaneous conversion to sinus rhythm were not considered to be untreated. Sinus rhythm was restored prior to the planned intervention, and these patients subsequently received anti-arrhythmic therapy for rhythm maintenance, in line with standard clinical practice. 

Patients were followed at six and 12 months to assess AF recurrence. Recurrence was defined as any documented episode of AF lasting >30 seconds, confirmed by a standard 12-lead ECG performed either during symptoms (e.g., palpitations, dyspnea) or during scheduled clinical evaluations. Continuation or modification of antiarrhythmic drugs, β-blockers, angiotensin-converting enzyme (ACE) inhibitors/angiotensin receptor blockers (ARBs), and anticoagulation therapy was recorded during each follow-up visit and included in the multivariable analysis as potential confounders. All patients attended their scheduled outpatient clinic visits at both follow-up time points. A 12-lead ECG was performed during each visit. According to the study protocol, patients were instructed to attend the hospital for a clinical evaluation and ECG to confirm a possible AF episode if they experienced symptoms between visits. During hospitalization, all patients underwent continuous 24-hour telemetry monitoring for at least 48 hours.

Statistical analysis

Quantitative variables were expressed as mean values±SD, while qualitative variables were expressed as absolute and relative frequencies. Receiver operating characteristic (ROC) curves were used in order to estimate the prognostic ability of LA size for having an AF episode at six or 12 months. Sensitivity, specificity, negative predictive value (NPV), and positive predictive value (PPV) were calculated for optimal cut-offs. The area under the curve (AUC) was also calculated. Optimal cut-off was identified via Youden’s index. Univariate logistic regression analysis was used to assess the association between participants’ characteristics and having an AF episode at six or 12 months. A bootstrap resampling procedure (1000 samples) was used to cross-validate AUCs. Multiple logistic regression analysis was used to find factors associated with having an AF episode at six or 12 months. Adjusted odds ratios (OR) with 95% confidence intervals (CIs) were computed from the results of the logistic regression analyses. 

All possible interactions were tested. A few missing values (two patients were lost to follow-up at six months and another one at 12 months) were present; thus, no replacement of the missing values was performed. Analyses were done using listwise deletion for handling missing data. All reported p-values are two-tailed. Statistical significance was set at p<0.05, and analyses were conducted by an independent statistician for minimizing the bias, using IBM SPSS Statistics for Windows, version 27.0 (Released 2019; IBM Corp., Armonk, New York, United States).

## Results

The study population consisted of 66 patients who were hospitalized due to AF, of whom 47 (71.2%) were male patients, and the mean age was 65.4±13.7 years. Their demographic and clinical characteristics are presented in Tables 1, 2. The majority of the sample had hypertension (n=49; 74.2%) and dyslipidemia (n=36; 54.5%), while 19 patients (28.8%) were smokers. Mean left ventricular ejection fraction (LVEF) was 52.3%±10.5%, and 51 patients (77.3%) had LVEF ≥50%. Mean LA size was 43.5±7.2 mm; 21 patients (31.8%) had LA <40 mm, and eight (12.1%) had LA >50 mm. AF was treated with electrical cardioversion (ECV) in 30 patients (46.9%), with propafenone in five patients (7.8%), with amiodarone in 12 patients (18.8%), with flecainide in 14 patients (21.9%), and automatic conversion in three patients (4.7%). 

**Table 1:** Demographical and clinical characteristics LVEF: left ventricular ejection fraction; LA: left atrium

Characteristics	Frequency	Percentage
Sex		
Male	47	71.2
Female	19	28.8
Smoking	19	28.8
Hypertension	49	74.2
Diabetes	15	22.7
Dyslipidemia	36	54.5
Alcohol	6	9.1
Stroke	4	6.1
Thyroid disorders	10	15.2
Coronary artery disease	18	27.3
Renal disease	3	4.5
Respiratory disease	4	6.1
LVEF (%)		
<40%	12	18.2
41-49%	3	4.5
>=50%	51	77.3
LA size (mm)		
<40	21	31.8
40-45	19	28.8
46-50	18	27.3
>50	8	12.1

**Table 2:** Patients’ age, LVEF, LA size, and number of comorbidities ^1^includes hypertension, diabetes, dyslipidemia, stroke, thyroid disorders, coronary artery disease, renal disease, and respiratory disease. LVEF: left ventricular ejection fraction; LA: left atrium

Characteristics	Mean ± SD
Age	65.4 ± 13.7
LVEF (%)	52.3 ± 10.5
LA size (mm)	43.5 ± 7.2
Number of comorbidities^1^	2.11 ± 1.22

The treatment that participants received at hospital discharge, at six and 12 months, as well as their AF episodes, are presented in Table 3. The most frequent symptom reported was palpitation, followed by dyspnea and weakness/ fatigue. At six months, 51.6% (n=33) of the sample had at least one AF episode, and at 12 months the corresponding percentage was 50.8% (n=32). B-blockers and antiarrhythmic therapy were the most common ways to deal with AF. There were no significant differences in patients' medication throughout the follow-up period (p>0.05). During hospitalization, 62.1% of patients had paroxysmal AF; however, by six and 12 months, most patients had persistent AF (60.6% and 68.8%, respectively). The change in AF type distribution (from paroxysmal to persistent) between consecutive timepoints was not significant (p>0.05). More specifically, 6/33 patients had their AF type changed from paroxysmal to persistent at 6 months compared to baseline, and 3/32 at 12 months compared to six months. LA size was not significantly associated with having AF type changed (p>0.05). 

**Table 3:** Treatment and AF episodes at hospital discharge, six months, and 12 months AF: atrial fibrillation; ACE: angiotensin-converting enzyme inhibitor; ARB: angiotensin receptor blocker

Parameters		At hospital discharge		6 Months		12 Months	
		Frequency	Percentage	Frequency	Percentage	Frequency	Percentage
AF Episodes (6 months)		-	-	33	51.6	32	50.8
Number of AF episodes	0	-	-	31	48.4	31	49.2
	1	-	-	24	37.5	30	47.6
	2	-	-	7	10.9	2	3.2
	3	-	-	1	1.6	0	0
	4	-	-	1	1.6	0	0
β-blockers		60	90.9	58	90.6	57	90.5
ACE inhibitors		15	22.7	10	15.6	10	15.9
ARBs		25	37.9	19	29.7	20	31.7
Calcium channel blockers		13	19.7	18	28.1	17	27.0
Diuretic		26	39.4	24	37.5	28	44.4
Statin		45	68.2	47	73.4	49	77.8
Antiarrhythmics		46	69.7	44	68.8	42	66.7
Type of Antiarrhythmic	Amiodarone (Class III)	20	43.5	28	66.7	30	71.4
	Flecainide (Class Ic)	20	43.5	11	26.2	12	28.6
	Propafenone (Class Ic)	6	13.0	3	7.1	0	0.0
	Sotalol (Class III)	0	0.0	0	0.0	0	0.0
Anticoagulants		61	92.4	57	90.5	58	92.1
Type of Anticoagulant	Xarelto (Rivaroxaban)	35	57.4	35	61.4	38	65.5
	Pradaxa (Dabigatran)	8	13.1	5	8.8	5	8.6
	Eliquis (Apixaban)	18	29.5	17	29.8	15	25.9
Type of AF	First diagnosed	0	0.0	0	0.0	1	3.1
	Paroxysmal	41	62.1	13	39.4	9	28.1
	Persistent	25	37.9	20	60.6	22	68.8
	Long-standing persistent	0	0.0	0	0.0	0	0.0
	Permanent	0	0.0	0	0.0	0	0.0
Symptoms	Palpitation	53	80.3	29	87.9	26	81.3
	Dyspnea	12	18.2	7	21.2	8	25.0
	Dizziness	3	4.5	1	3.0	2	6.3
	Chest pain	4	6.1	3	9.1	1	3.1
	Syncope	1	1.5	0	0.0	0	0.0
	Weakness/ Fatigue	7	10.6	2	6.1	5	15.6

None of the demographic and clinical characteristics were found to be significantly associated with experiencing AF episodes at six and 12 months, p>0.05 (Table 4).

**Table 4:** Association of demographic and clinical characteristics with experiencing AF episodes at six and 12 months ‡Student’s t-test; +Pearson’s χ2 test; ++Fisher’s exact test AF: atrial fibrillation

Parameters		AF episodes (6 months)		p-value	AF episodes (12 months)		p-value
		No	Yes		No	Yes	
		Frequency (Percentage)	Frequency (Percentage)		Frequency (Percentage)	Frequency (Percentage)	
Age (years), mean±SD		64±11.4	66.2±15.8	0.542‡	64.3±13.7	66.8±13.2	0.466‡
Sex	Male	21 (46.7)	24 (53.3)	0.663+	21 (47.7)	23 (52.3)	0.721+
	Female	10 (52.6)	9 (47.4)		10 (52.6)	9 (47.4)	
Smoking	Yes	11 (57.9)	8 (42.1)	0.325+	6 (31.6)	13 (68.4)	0.066+
	No	20 (44.4)	25 (55.6)		25 (56.8)	19 (43.2)	
Hypertension	Yes	23 (48.9)	24 (51.1)	0.894+	22 (46.8)	25 (53.2)	0.514+
	No	8 (47.1)	9 (52.9)		9 (56.3)	7 (43.8)	
Diabetes	Yes	9 (64.3)	5 (35.7)	0.179+	6 (42.9)	8 (57.1)	0.590+
	No	22 (44)	28 (56)		25 (51)	24 (49)	
Dyslipidemia	Yes	18 (51.4)	17 (48.6)	0.599+	18 (51.4)	17 (48.6)	0.693+
	No	13 (44.8)	16 (55.2)		13 (46.4)	15 (53.6)	
Alcohol	Yes	2 (33.3)	4 (66.7)	0.673++	3 (50)	3 (50)	>0.999++
	No	29 (50)	29 (50)		28 (49.1)	29 (50.9)	
Stroke	Yes	2 (50)	2 (50)	>0.999++	4 (100)	0 (0)	0.053++
	No	29 (48.3)	31 (51.7)		27 (45.8)	32 (54.2)	
Thyroid disorders	Yes	2 (20)	8 (80)	0.083++	4 (40)	6 (60)	0.732++
	No	29 (53.7)	25 (46.3)		27 (50.9)	26 (49.1)	
Coronary artery disease	Yes	9 (52.9)	8 (47.1)	0.665+	9 (52.9)	8 (47.1)	0.718+
	No	22 (46.8)	25 (53.2)		22 (47.8)	24 (52.2)	
Renal disease	Yes	1 (50)	1 (50)	>0.999++	2 (100)	0 (0)	0.238++
	No	30 (48.4)	32 (51.6)		29 (47.5)	32 (52.5)	
Respiratory disease	Yes	2 (50)	2 (50)	>0.999++	1 (25)	3 (75)	0.613++
	No	29 (48.3)	31 (51.7)		30 (50.8)	29 (49.2)	

Treatment and symptoms at hospital discharge and at six months were not significantly associated with having an AF episode at or before the six-month follow-up, p>0.05 (Table 5).

**Table 5:** Association of patients' treatment and symptoms at hospital discharge and at six months with experiencing AF episodes at six months* +Pearson’s χ2 test; ++Fisher’s exact test *Two patients were lost to follow-up at six months and no replacement of the missing values was performed AF: atrial fibrillation; ACE: angiotensin-converting enzyme inhibitor; ARB: angiotensin receptor blocker

Parameters		AF episodes (6 months)		p-value
		No	Yes	
		Frequency (percentage)	Frequency (percentage)	
At hospital discharge				
β-blockers	Yes	27 (46.6)	31 (53.4)	0.419++
	No	4 (66.7)	2 (33.3)	
ACE inhibitor	Yes	6 (40)	9 (60)	0.455+
	No	25 (51)	24 (49)	
ARB	Yes	11 (45.8)	13 (54.2)	0.747+
	No	20 (50)	20 (50)	
CCB	Yes	4 (33.3)	8 (66.7)	0.245+
	No	27 (51.9)	25 (48.1)	
Diuretics	Yes	11 (44)	14 (56)	0.570+
	No	20 (51.3)	19 (48.7)	
Statin	Yes	22 (51.2)	21 (48.8)	0.532+
	No	9 (42.9)	12 (57.1)	
Antiarrhythmics	Yes	23 (51.1)	22 (48.9)	0.510+
	No	8 (42.1)	11 (57.9)	
Anticoagulants	Yes	29 (49.2)	30 (50.8)	>0.999++
	No	2 (40)	3 (60)	
Type of AF	First diagnosed	0 (0)	0 (0)	0.175+
	Paroxysmal	22 (55)	18 (45)	
	Persistent	9 (37.5)	15 (62.5)	
	Long-standing persistent	0 (0)	0 (0)	
	Permanent	0 (0)	0 (0)	
Palpitation	No	6 (50)	6 (50)	0.904+
	Yes	25 (48.1)	27 (51.9)	
Dyspnea	No	27 (51.9)	25 (48.1)	0.245+
	Yes	4 (33.3)	8 (66.7)	
Dizziness	No	30 (48.4)	32 (51.6)	>0.999++
	Yes	1 (50)	1 (50)	
Chest pain	No	28 (46.7)	32 (53.3)	0.347++
	Yes	3 (75)	1 (25)	
Syncope	No	30 (47.6)	33 (52.4)	0.484++
	Yes	1 (100)	0 (0)	
Weakness/ Fatigue	No	28 (49.1)	29 (50.9)	>0.999++
	Yes	3 (42.9)	4 (57.1)	
Treatment	Amiodarone	6 (50)	6 (50)	0.889++
	Propafenone	2 (40)	3 (60)	
	Flecainide	7 (53.8)	6 (46.2)	
	Electrical cardioversion	12 (41.4)	17 (58.6)	
	Spontaneous conversion	2 (66.7)	1 (33.3)	
6 months				
b-blockers	Yes	28 (48.3)	30 (51.7)	>0.999++
	No	3 (50)	3 (50)	
ACE inhibitor	Yes	4 (40)	6 (60)	0.734++
	No	27 (50)	27 (50)	
ARB	Yes	9 (47.4)	10 (52.6)	>0.999++
	No	22 (48.9)	23 (51.1)	
CCB	Yes	8 (44.4)	10 (55.6)	0.689+
	No	23 (50)	23 (50)	
Diuretic	Yes	8 (33.3)	16 (66.7)	0.061+
	No	23 (57.5)	17 (42.5)	
Statin	Yes	24 (51.1)	23 (48.9)	0.485+
	No	7 (41.2)	10 (58.8)	
Antiarrhythmics	Yes	20 (45.5)	24 (54.5)	0.479+
	No	11 (55)	9 (45)	
Anticoagulants	Yes	27 (47.4)	30 (52.6)	>0.999++
	No	3 (50)	3 (50)	
Type of AF	First diagnosed	0 (0)	0 (0)	-
	Paroxysmal	0 (0)	13 (100)	
	Persistent	0 (0)	20 (100)	
	Long-standing persistent	0 (0)	0 (0)	
	Permanent	0 (0)	0 (0)	
Symptoms	No	0 (0)	0 (0)	-
	Yes	0 (0)	33 (100)	
Palpitation	No	0 (0)	4 (100)	-
	Yes	0 (0)	29 (100)	
Dyspnea	No	0 (0)	26 (100)	-
	Yes	0 (0)	7 (100)	
Dizziness	No	0 (0)	32 (100)	-
	Yes	0 (0)	1 (100)	
Chest pain	No	0 (0)	30 (100)	-
	Yes	0 (0)	3 (100)	
Syncope	No	0 (0)	33 (100)	-
	Yes	0 (0)	0 (0)	
Weakness/ Fatigue	No	0 (0)	31 (100)	-
	Yes	0 (0)	2 (100)	

On the contrary, it was found that having a persistent AF at the hospital was significantly associated with a greater percentage of having an AF episode at 12 months, compared to a paroxysmal AF at the hospital (66.7% (n=16) vs 41% (n=16); p=0.048) (Table 6). Also, taking ACE inhibitors at six months was significantly associated with a lower percentage of having an AF episode at 12 months (20% (n=2) vs 56.6% (n=30); p=0.043).

**Table 6:** Association of patients' treatment and symptoms at hospital discharge, at six months, and at 12 months with experiencing AF episodes at 12 months* +Pearson’s χ2 test; ++Fisher’s exact test *two patients were lost to follow-up at six months and another one at 12 months and no replacement of the missing values was performed AF: atrial fibrillation; ACE: angiotensin-converting enzyme inhibitor; ARB: angiotensin receptor blocker

Parameters		AF episodes (12 months)		P
		No	Yes	
		Frequency (Percentage)	Frequency (Percentage)	
At hospital discharge				
b-blockers	Yes	28 (49.1)	29 (50.9)	>0.999++
	No	3 (50)	3 (50)	
ACE inhibitors	Yes	8 (53.3)	7 (46.7)	0.714+
	No	23 (47.9)	25 (52.1)	
ARB	Yes	10 (41.7)	14 (58.3)	0.348+
	No	21 (53.8)	18 (46.2)	
CCB	Yes	6 (50)	6 (50)	0.951+
	No	25 (49)	26 (51)	
Diuretics	Yes	12 (48)	13 (52)	0.877+
	No	19 (50)	19 (50)	
Statin	Yes	21 (50)	21 (50)	0.859+
	No	10 (47.6)	11 (52.4)	
Antiarrhythmics	Yes	21 (47.7)	23 (52.3)	0.721+
	No	10 (52.6)	9 (47.4)	
Anticoagulants	Yes	27 (46.6)	31 (53.4)	0.196++
	No	4 (80)	1 (20)	
Type of AF	First diagnosed	0 (0)	0 (0)	0.048++
	Paroxysmal	23 (59)	16 (41)	
	Persistent	8 (33.3)	16 (66.7)	
	Long-standing persistent	0 (0)	0 (0)	
	Permanent	0 (0)	0 (0)	
Palpitation	No	7 (58.3)	5 (41.7)	0.482+
	Yes	24 (47.1)	27 (52.9)	
Dyspnea	No	24 (47.1)	27 (52.9)	0.482+
	Yes	7 (58.3)	5 (41.7)	
Dizziness	No	30 (49.2)	31 (50.8)	>0.999++
	Yes	1 (50)	1 (50)	
Chest pain	No	28 (47.5)	31 (52.5)	0.355++
	Yes	3 (75)	1 (25)	
Syncope	No	30 (48.4)	32 (51.6)	0.492++
	Yes	1 (100)	0 (0)	
Weakness/ Fatigue	No	28 (50)	28 (50)	>0.999++
	Yes	3 (42.9)	4 (57.1)	
Treatment	Amiodarone	6 (50)	6 (50)	0.902++
	Propafenone	1 (25)	3 (75)	
	Flecainide	7 (53.8)	6 (46.2)	
	Electrical cardioversion	14 (48.3)	15 (51.7)	
	Spontaneous conversion	2 (66.7)	1 (33.3)	
6 months				
β-blockers	Yes	27 (47.4)	30 (52.6)	0.426++
	No	4 (66.7)	2 (33.3)	
ACE inhibitors	Yes	8 (80)	2 (20)	0.043++
	No	23 (43.4)	30 (56.6)	
ARB	Yes	8 (42.1)	11 (57.9)	0.459+
	No	23 (52.3)	21 (47.7)	
CCB	Yes	8 (44.4)	10 (55.6)	0.633+
	No	23 (51.1)	22 (48.9)	
Diuretic	Yes	11 (45.8)	13 (54.2)	0.674+
	No	20 (51.3)	19 (48.7)	
Statin	Yes	22 (46.8)	25 (53.2)	0.514+
	No	9 (56.3)	7 (43.8)	
Antiarrhythmics	Yes	19 (44.2)	24 (55.8)	0.243+
	No	12 (60)	8 (40)	
Anticoagulants	Yes	27 (47.4)	30 (52.6)	0.426++
	No	4 (66.7)	2 (33.3)	
Type of AF	Firstdiagnosed	0 (0)	0 (0)	0.461++
	Paroxysmal	5 (38.5)	8 (61.5)	
	Persistent	5 (25)	15 (75)	
	Long-standing persistent	0 (0)	0 (0)	
	Permanent	0 (0)	0 (0)	
Symptoms	No	0 (0)	0 (0)	-
	Yes	10 (30.3)	23 (69.7)	
Palpitation	No	2 (50)	2 (50)	0.567++
	Yes	8 (27.6)	21 (72.4)	
Dyspnea	No	9 (34.6)	17 (65.4)	0.397++
	Yes	1 (14.3)	6 (85.7)	
Dizziness	No	10 (31.3)	22 (68.8)	>0.999++
	Yes	0 (0)	1 (100)	
Chest pain	No	8 (26.7)	22 (73.3)	0.212++
	Yes	2 (66.7)	1 (33.3)	
Syncope	No	10 (30.3)	23 (69.7)	-
	Yes	0 (0)	0 (0)	
Weakness/ Fatigue	No	10 (32.3)	21 (67.7)	>0.999++
	Yes	0 (0)	2 (100)	
12 months				
β-blockers	Yes	27 (47.4)	30 (52.6)	0.426++
	No	4 (66.7)	2 (33.3)	
ACE inhibitors	Yes	6 (60)	4 (40)	0.509++
	No	25 (47.2)	28 (52.8)	
ARB	Yes	8 (40)	12 (60)	0.319+
	No	23 (53.5)	20 (46.5)	
CCB	Yes	7 (41.2)	10 (58.8)	0.438+
	No	24 (52.2)	22 (47.8)	
Diuretics	Yes	13 (46.4)	15 (53.6)	0.693+
	No	18 (51.4)	17 (48.6)	
Statin	Yes	22 (44.9)	27 (55.1)	0.201+
	No	9 (64.3)	5 (35.7)	
Antiarrhythmics	Yes	19 (45.2)	23 (54.8)	0.373+
	No	12 (57.1)	9 (42.9)	
Anticoagulants	Yes	27 (46.6)	31 (53.4)	0.196++
	No	4 (80)	1 (20)	
Type of AF	First diagnosed	0 (0)	1 (100)	-
	Paroxysmal	0 (0)	9 (100)	
	Persistent	0 (0)	22 (100)	
	Long-standing persistent	0 (0)	0 (0)	
	Permanent	0 (0)	0 (0)	
Symptoms	No	0 (0)	1 (100)	-
	Yes	0 (0)	31 (100)	
Palpitation	No	0 (0)	6 (100)	-
	Yes	0 (0)	26 (100)	
Dyspnea	No	0 (0)	24 (100)	-
	Yes	0 (0)	8 (100)	
Dizziness	No	0 (0)	30 (100)	-
	Yes	0 (0)	2 (100)	
Chest pain	No	0 (0)	31 (100)	-
	Yes	0 (0)	1 (100)	
Syncope	No	0 (0)	32 (100)	-
	Yes	0 (0)	0 (0)	
Weakness/ Fatigue	No	0 (0)	27 (100)	-
	Yes	0 (0)	5 (100)	

**Figure 1:**
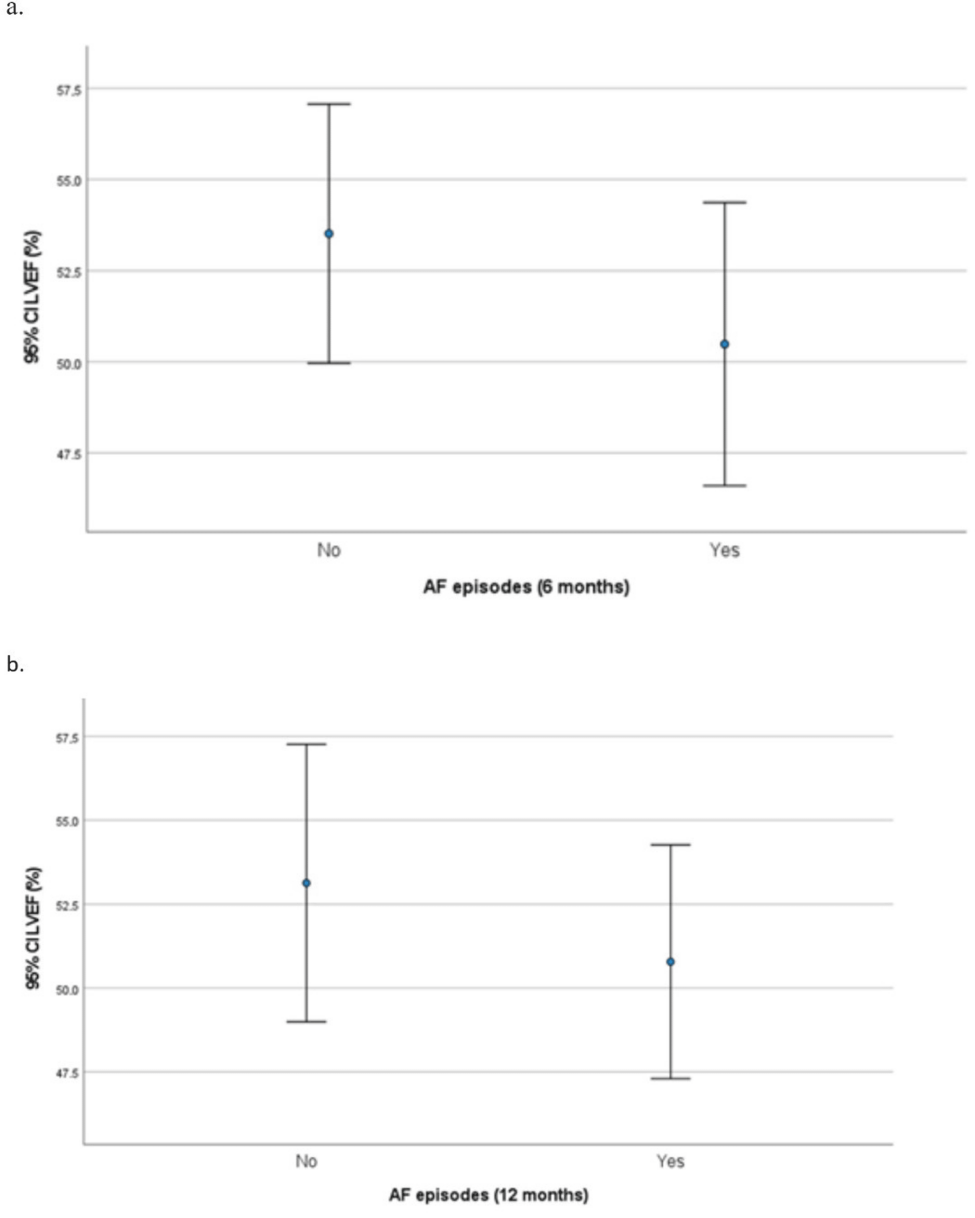
Left ventricular ejection fraction (LVEF) according to atrial fibrillation (AF) recurrence at (a) six months (a) and (b) 12 months.

**Figure 2:**
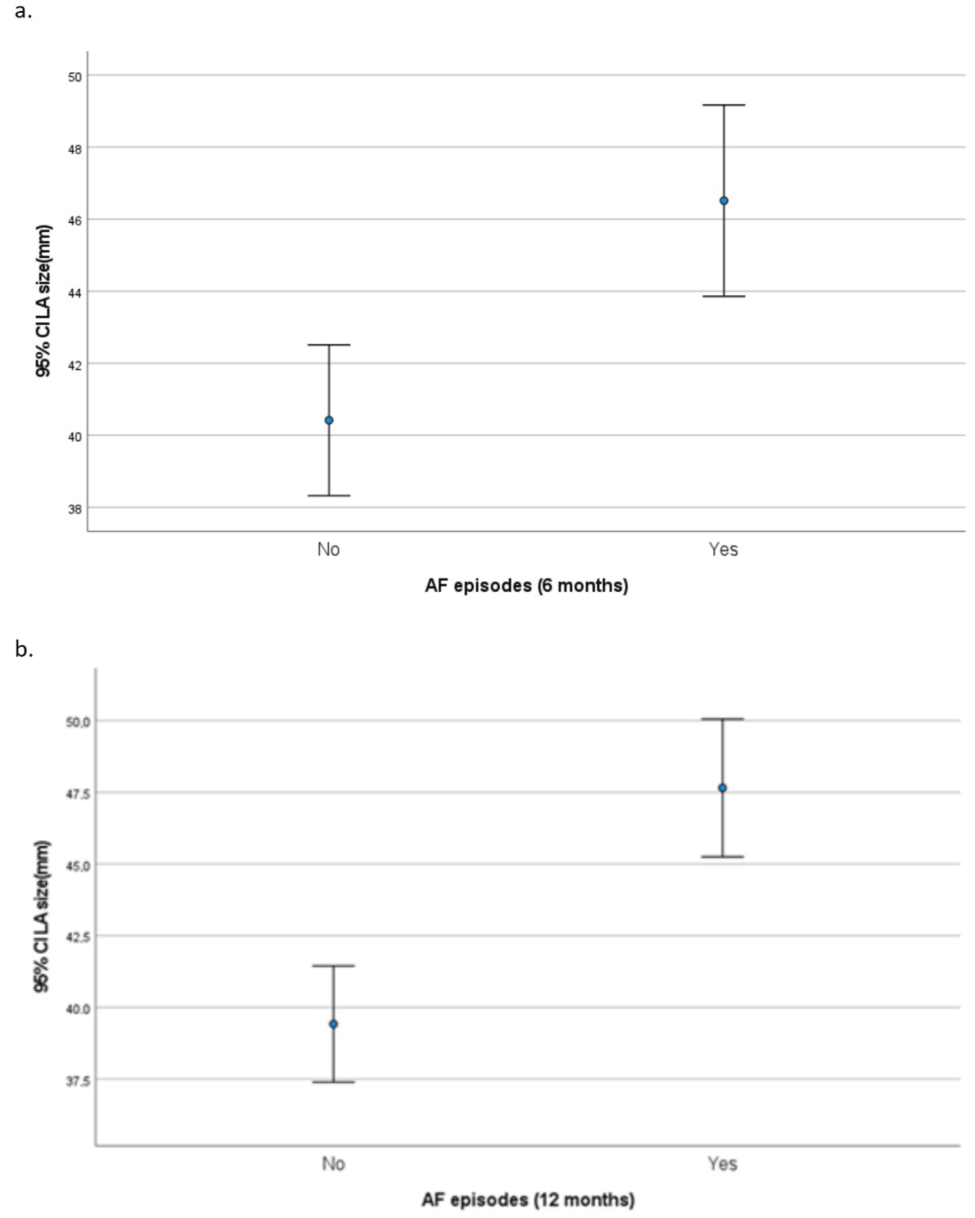
Left atrial (LA) diameter according to atrial fibrillation (AF) recurrence at (a) six months and (b) 12 months

The association of LVEF and LA size with experiencing AF episodes at six and 12 months is presented in Table 7. Mean LVEF values were not found to be associated with experiencing AF episodes at six and 12 months (Figure 1). However, having LVEF<50% was significantly associated with a greater percentage of having an AF episode at six months. LA size was found to be significantly greater in patients who had an AF episode at six or 12 months (Figure 2).

**Table 7:** Association of LVEF and LA size with experiencing AF episodes at six months (N=64) and 12 months (N=63)* *two patients were lost to follow-up at six months and another one at 12 months and no replacement of the missing values was performed LVEF: left ventricular ejection fraction; LA: left atrium; AF: atrial fibrillation

Parameters				AF episodes (6 months)		OR (95% CI)+	p-value	AF episodes (12 months)		OR (95% CI)	p-value
				No	Yes			No	Yes		
				Frequency (Percentage)	Frequency (Percentage)			Frequency (Percentage)	Frequency (Percentage)		
LVEF (%), mean±SD				53.5±9.7	50.5±10.9	0.97 (0.92 – 1.02)	0.246	53.1±11.3	50.8±9.7	0.98 (0.93 – 1.03)	0.373
LVEF (%)	<50%		3 (20)		12 (80)	Reference		6 (40)	9 (60)	0.61 (0.19 – 1.99)	0.416
	>=50%		28 (57.1)		21 (42.9)	0.19 (0.05 – 0.75	0.018	25 (52.1)	23 (47.9)		
LA size (mm), mean (SD)				40.4±5.7	46.5±7.5	1.15 (1.05 – 1.27)	0.002	39.4±5.5	47.7±6.7	1.27 (1.12 – 1.43)	<0.001
LA size (mm)		<40	15 (75)		5 (25)	Reference		17 (85)	3 (15)	Reference	
		40-45	10 (55.6)		8 (44.4)	2.40 (0.61 – 9.49)	0.212	8 (47.1)	9 (52.9)	0.03 (0.00 – 0.29)	0.003
		46-50	4 (22.2)		14 (77.8)	10.5 (2.34 – 47.20)	0.002	5 (27.8)	13 (72.2)	0.16 (0.02 – 1.61)	0.120
		>50	2 (25)		6 (75)	9.00 (1.36 – 59.78)	0.023	1 (12.5)	7 (87.5)	0.37 (0.04 – 3.84)	0.406
LA size (mm)		<40	15 (75)		5 (25)	Reference		17 (85)	3 (15)	Reference	
		40-50	14 (38.9)		22 (61.1)	4.71 (1.40 – 15.87)	0.012	13 (37.1)	22 (62.9)	9.59 (2.35 – 39.12)	0.002
		>50	2 (25)		6 (75)	9.00 (1.36 – 59.78)	0.023	1 (12.5)	7 (87.5)	39.67 (3.50 – 449.81)	0.003

**Figure 3:**
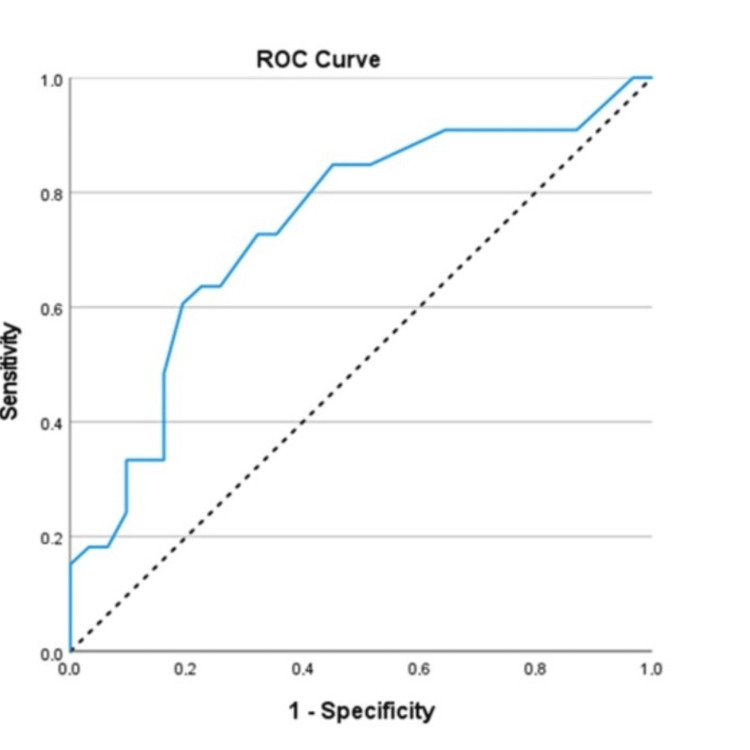
ROC curve for the prognostic ability of LA for having an AF episode at six months ROC: receiver operator characteristic; LA: left atrium; AF: atrial fibrillation

ROC analysis demonstrated that the LA size had a significant prognostic ability for having an AF episode at six months (n=64; AUC=0.74; 95%CI: 0.62 - 0.86; p=0.001) (Figure 3). Optimal cut-off was found to be 42.5. Sensitivity was 72.7%, specificity 67.7%, PPV 70.6%, and NPV 70%. Also, participants with LA size >42.5 had 5.60 times greater probability of having an AF episode at six months (70.6% vs 30%; OR=5.60; 95%CI: 1.91 - 16.40; p=0.002). 

**Figure 4:**
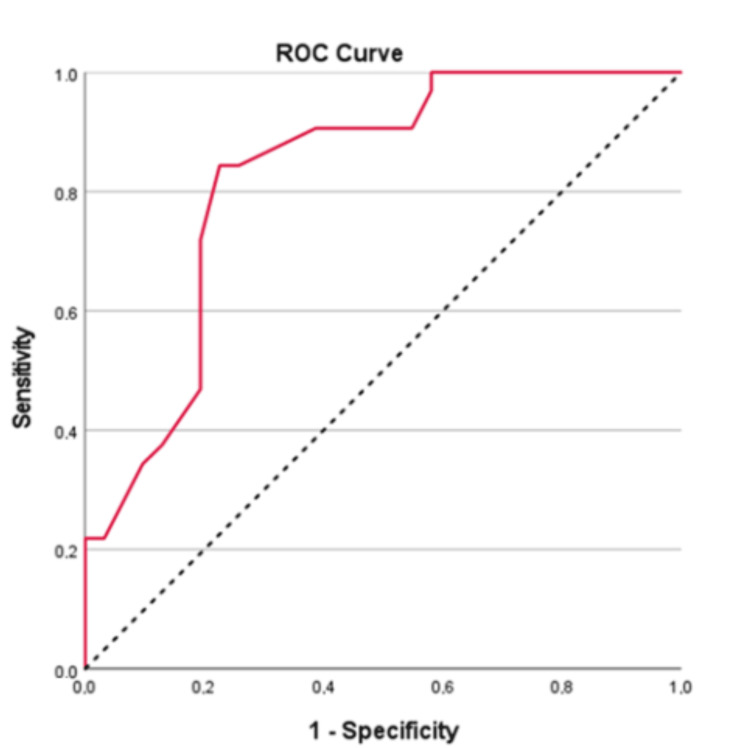
ROC curve for the prognostic ability of LA for having an AF episode at 12 months ROC: receiver operator characteristic; LA: left atrium; AF: atrial fibrillation

The ROC analysis showed that LA size had a significant prognostic ability for having an AF episode at 12 months (n=63; AUC=0.83; 95%CI: 0.72 - 0.93; p<0.001) (Figure 4). Optimal cut-off was also found to be 42.5. Sensitivity was 84.4%, specificity 77.4%, PPV 79.4%, and NPV 82.8%. Also, participants with LA size >42.5 had 18.5 times greater probability of having an AF episode at 12 months (79.4% vs 17.2%; OR=18.51; 95%CI: 5.19 - 66.10; p<0.001). There was no significant difference in the six- and 12-month predictive performance (p>0.05). After the bootstrap procedure, the AUC was stable at both six and 12 months.

From multivariate logistic regression, it emerged that having LA size>42.5 was significantly associated with having an AF episode at six (OR=7.55; 95%CI: 2.22 - 25.66; p=0.001) and 12 months (OR=41.97; 95%CI: 7.37 - 239.07; p<0.001), after adjusting simultaneously for age, gender, smoking, alcohol and number of comorbidities (Table 8). Also, patients who smoked had a significantly greater probability of having an AF episode at 12 months (OR=12.9; 95%CI: 1.70 - 97.75; p=0.001). All possible interactions were tested, but no significant results emerged.

**Table 8:** Multivariate logistic regression results with having an AF episode at six (n=64) and 12 months (n=63) as dependent variables Note: All model assumptions for logistic regression (e.g., linearity, absence of multicollinearity) were verified and satisfied ^1^includes hypertension, diabetes, dyslipidemia, stroke, thyroid disorders, coronary artery disease, renal disease and respiratory disease LA: left atrium; AF: atrial fibrillation

Dependent variable	Independent variable	OR (95% CI)+	P
AF episode at 6 months (Hosmer-Lemeshow test p=0.984; Nagelkerke R²=0.29)	LA size (>42,5 vs <42,5)	7.55 (2.22 ─ 25.66)	0.001
	Age	1.01 (0.96 ─ 1.06)	0.746
	Sex (Female vs male)	0.63 (0.17 ─ 2.27)	0.480
	Smoking (Yes vs No)	0.29 (0.07 ─ 1.27)	0.100
	Alcohol (Yes vs No)	2.87 (0.3 ─ 27.56)	0.362
	Number of comorbidities^1^	0.72 (0.41 ─ 1.25)	0.239
AF episode at 12 months (Hosmer-Lemeshow test p=0.909; Nagelkerke R²=0.59)	LA size (>42,5 vs <42,5)	41.97 (7.37 ─ 239.07)	<0.001
	Age	1.06 (0.98 ─ 1.16)	0.158
	Sex (Female vs male)	0.55 (0.1 ─ 3.02)	0.491
	Smoking (Yes vs No)	12.9 (1.7 ─ 97.75)	0.013
	Alcohol (Yes vs No)	0.08 (0.01 ─ 1.07)	0.056
	Number of comorbidities^1^	0.55 (0.29 ─ 1.06)	0.075

## Discussion

This prospective observational study demonstrates a significant association between increased LA diameter and the recurrence of AF at both six and 12 months following rhythm control interventions. An LA diameter >42.5 mm was found to be a strong and independent predictor of AF recurrence, with adjusted odds ratios of 7.55 and 41.97 at 6 and 12 months, respectively. While prior studies have reported an association between left atrial enlargement and atrial fibrillation recurrence, this prospective study adds value by confirming the prognostic utility of left atrial diameter in a real-world, regional hospital setting. By focusing on a simple, widely available echocardiographic parameter, our findings demonstrate the clinical applicability of LA diameter for risk stratification after rhythm restoration, particularly in settings where advanced imaging techniques are not routinely accessible.

The Framingham Heart Study was among the first to identify LA enlargement as a predictor of incident AF in the general population [13]. Other studies, such as that by Tsang et al. [14], have confirmed that increased LA volume is an independent risk factor for both the development and recurrence of AF after cardioversion.

Our findings are consistent with those of Gertz et al., who showed that patients with mitral regurgitation (MR) undergoing catheter ablation for AF had larger LA dimensions (4.5 cm vs. 4.1 cm) and a higher prevalence of persistent AF (7% vs. 28%) compared with those without MR [15]. Similarly, many studies have reported that a LA diameter greater than 45-50 mm is an independent predictor of AF recurrence, whether assessed by TTΕ or TEE [16]. In particular, Pappone et al. evaluated 589 patients undergoing pulmonary vein isolation (PVI) and demonstrated that an LA diameter exceeding 45 mm independently predicted AF recurrence in both paroxysmal and persistent forms of the arrhythmia [17]. In our study, patients with LA diameter >42.5 mm had recurrence rates exceeding 80% at 12 months, and ROC analysis confirmed excellent discriminatory power (AUC = 0.83), underscoring the reproducibility and clinical utility of this echocardiographic marker. Further support for our observations is provided by the CABANA Imaging Substudy conducted by Rettmann et al., which demonstrated that a rhythm control strategy via catheter ablation leads to a more pronounced reversal of structural remodeling of the left atrium compared to antiarrhythmic drug therapy [18]. Specifically, within approximately 100 days post-intervention, their patients who underwent ablation showed a greater reduction in left atrial volume index (−52.9%) compared to those receiving antiarrhythmic medications (−40.0%). These findings highlight the significance of early structural remodeling following intervention and reinforce the diagnostic and prognostic value of left atrial diameter, as reflected in our own study, where a diameter >42.5 mm was associated with markedly high recurrence rates of atrial fibrillation at 12 months.

In contrast to LA size, LVEF was not significantly associated with AF recurrence in our cohort. Consistent with our findings, a meta-analysis including 13 studies and 2.825 patients demonstrated that elevated LA diameter was significantly associated with AF recurrence after catheter ablation (mean difference (MD) = 2.19, 95% CI: 1.63-2.75, P < .001). In contrast, baseline LVEF showed no significant relationship with AF recurrence (MD = −0.91, 95% CI: −1.18 to 1.67, P = 14). These findings suggest that LA size, rather than ventricular systolic function, plays a more decisive role in predicting post-ablation AF recurrence, particularly in patients with preserved or mildly reduced LVEF [19]. 

In addition, in a meta-analysis of 21 studies including over 2.800 patients, Bajraktari et al. reported that patients with AF recurrence had significantly larger LA diameters and volumes, as well as reduced atrial strain and emptying fraction [20]. An LA diameter ≥50 mm was among the strongest predictors of recurrence (OR = 2.75; 95%CI 1.66-4.56; P < 0.001), emphasizing the prognostic importance of left atrial size in post-ablation outcomes, which was consistent with our study.

Another important finding in our study is the association between smoking and AF recurrence at 12 months (OR = 12.9). Smoking has been implicated in AF pathogenesis through mechanisms such as oxidative stress, atrial fibrosis, and autonomic imbalance [21]. Our data reinforce previous findings, such as those by Chamberlain et al. [22], who reported higher AF incidence among smokers even after adjusting for comorbidities.

Interestingly, patients who were receiving ACE inhibitors at 12 months demonstrated lower recurrence rates. While not the primary endpoint, this observation echoes earlier evidence suggesting that renin-angiotensin system (RAS) inhibition may attenuate atrial fibrosis and reduce AF recurrence, especially in patients with hypertension or structural heart disease [23].

Limitations

This study has several limitations. First, it was a single-center study with a relatively small sample size, which may limit the generalizability of the findings. Although echocardiographic measurements were performed using a standardized protocol by a single operator, assessment of LA diameter remains operator-dependent and subject to measurement variability. Second, follow-up assessment of AF recurrence was based on symptom-driven and scheduled ECG recordings, without continuous rhythm monitoring or systematic Holter use, which may have led to underestimation of asymptomatic AF episodes. Despite multivariable adjustment, residual confounding cannot be excluded, and data collection was not blinded, introducing potential observational bias. In addition, volumetric imaging techniques and LA functional parameters, such as LA strain, were not available for the validation of LA diameter measurements.

Future studies should include larger, multicentre cohorts and continuous rhythm monitoring to enable a comparative assessment of LA volumetric and functional indices. This will help to further refine risk stratification for AF recurrence.

Strengths 

This study’s main strengths include its prospective design with predefined follow-up at six and 12 months and the use of standardized rhythm control strategies according to ESC guidelines. LA diameter was measured using a standardized echocardiographic protocol by a single blinded operator, reducing measurement variability. AF recurrence was defined using clear, clinically relevant criteria and confirmed by ECG. Multivariable analysis allowed adjustment for key confounders, while the focus on LA diameter enhances the clinical applicability of the findings, particularly in real-world and resource-limited settings. This study may stimulate further larger-scale studies and meta-analyses, contributing to more individualized rhythm control strategies and the avoidance of unnecessary interventions.

## Conclusions

LA diameter >42.5 mm was a strong and independent predictor of AF recurrence at six and 12 months. However, this association is predictive rather than causal. Incorporating LA diameter into clinical risk stratification may guide individualized rhythm control strategies and early intervention for patients at higher risk while emphasizing modifiable factors such as smoking. Further validation in larger, multicenter cohorts, along with exploration of additional echocardiographic parameters (e.g., LA volume and strain), is warranted to refine prognostic accuracy.
